# Supplementary material for: Patterns of Intron Gain and Loss in Fungi
Source: PLoS Biol. 2004 Nov 30;2(12):e422. doi: 10.1371/journal.pbio.0020422 (PMC532390; doi:10.1371/journal.pbio.0020422)
Supplement: Table S1 — Also available at http://genes.mit.edu/NielsenEtAl/. (4.3 MB ZIP). [file pbio.0020422.st001.zip › NielsenEtAl/html/133.html]

AN9522.1.NCU04099.1.MG09516.1.FG07173.1


```
 CLUSTAL W (1.82) Multiple Sequence Alignments - Introns Inserted


Sequence 1: MG09516.1	403 aa
Sequence 2: FG07173.1	400 aa
Sequence 3: NCU04099.1	400 aa
Sequence 4: AN9522.1	380 aa
Alignment Length: 414 aa
Number Identitical Residues: 181 aa
Alignment Score (without introns) 8871


MG09516.1 	MATPPPPLAGLKVLEFAGLAPG~PFAGMFLADAGASVLRLDRPG----RAPTEDMLARRK
NCU04099.1	-MVGQPPLTGLKVLEFAGLAPG1PFAGLLLADAGASVLRIDRAVSGPVARQVPDQLTRHK
FG07173.1 	-MTSPPPLTGINVLEFAGLAPG1PYAGMLLADAGASVLRIDRASS-DVSRPTADVLARHK
AN9522.1  	-MPTQLPLAGLRVVELAGLAPG1PFAGLLLADYGASVLRIDRPKS-----ISADQLTRGK
          	      **:*:.*:*:****** *:**::*** ******:**. :        * *:* *

MG09516.1 	ASIAVDLKSAGGVALAAKLAGHVDVLIDPFRPGVLERLGLGP-DVLR--AANPRLIYARI
NCU04099.1	SSLVVDLKSPSGIALIKSLAAVSDVLIDPYRPGVLEKLGLGP-SVLCSDECNPRLIYARL
FG07173.1 	ASISVDLKSERGIALIKRLAANADVLIDPFRPGVLEKLGLGP-DVLC--NLNKRLIYSRM
AN9522.1  	SSIILDLRDPASHKLLLELLSNADVLIDPYRPGVLERLGLSPKDVLL--KRNPRLIVARM
          	:*: :**:.  .  *   * .  ******:******:***.*..**     * *** :*:

MG09516.1 	AGFRRDGKYADMAGHDINYLAVSGALSMLGRKGEKPTPPWNILADFAGGGANLFQGILLA
NCU04099.1	TGFRRDGRFATMAGHDINYLAVSGVLSLLGRKGEKPTPPINILGDFAGGGAVLFQGILLA
FG07173.1 	TGFRRDGKYALMAGHDINYLAVSGTLGMLGREGEKPHPPMNILADFAGGGAILFQGILMA
AN9522.1  	TGFRRDGKYKDMAGHDINYIAVSGVLSMLGRAGEKPYAPGNILGDFAGGGAVCFQGILLA
          	:******::  ********:****.*.:*** **** .* ***.*******  *****:*

MG09516.1 	LAARGANGGKGQVVEANMVDGSAYLATFPRMALKTILGGAPRGENMLDTGCPFYDTYETA
NCU04099.1	LAAR-ERTGKGQVVEANIVDGASYLATFNRFALKTAVGNAPRGENLLDTGCPYYDTYETA
FG07173.1 	VISR-QSSGLGQVVEANMVDGSSHLTTFPRMGLKTPIGNRGRGKNLLDGAAPFYDTYETS
AN9522.1  	LLSR-ATTGRGQVVEANMVDGSAYLAAMPRLNMQTPLWSGPRGTNMLDGGSPFYDTYETK
          	: :*    * *******:***:::*::: *: ::* : .  ** *:** ..*:****** 

MG09516.1 	DSGSYMAVGALEPQFFAVLMRGLGLHGQGWEENRYDRDHWPELRTLFEKTFKSRTRAEWE
NCU04099.1	D-GKYIAVGALEPQFFKELVKGLGLEGQGWEERRGDKENWPELRRVLEHKFKTKTRREWE
FG07173.1 	D-GKYMAVGALEPQFFAALLEGLGLVNQGWEKDQQDRTRWPELRKLMTDTFRNKNRAEWE
AN9522.1  	DTGKYFSVGALEPQFYAALIKGLGFQPDELP-SREDRSNWPALRAAFTKRFKEKTRAEWE
          	*:*.*::********:  *:.***:  :     : *: .** **  : . *: :.* ***

MG09516.1 	NVFDGTDACCTPVLTFPELEEGRTSSSRPREGDQRPPVGLRDTPLLAVHQAV--QDPATQ
NCU04099.1	DIFDGTDACCTAVFEYGEMERERER----LEGDQRPVVTLRETPCLALRSDA---KDASH
FG07173.1 	AIFDGTDACCTPVLEYGELETDPD-----REGDQRPAVTLRETPCLAVKEGVGHVDPIRF
AN9522.1  	AVFDGTDACATPVLEQSELREAGFE--------QRPIVHLSDTPARPIAADD--------
          	 :*******.*.*:   *:.             *** * * :**  .:            

MG09516.1 	GQGAGVPGGGYEAIHLQPGQGGEELLGQWLGWAKGREFEAVD-GALVLNDK-SKL
NCU04099.1	GQGPGVKGEAYVGIPLKPGKGGESVVEKWLGWKKGKEFDVLN-GSAVAIK--SRL
FG07173.1 	GQGPGVPGDGYKANLLKPGAGGEEVLRQWMGWENEREYALQL-GGLVLKEA-SKL
AN9522.1  	--------GAWAGGILKPGTEGRETLKTWLGWEQGREYGVRSDGALVQTDGKSKL
          	         .: .  *:**  *.. :  *:** : :*:    .*. *  . .*:*
```
